# Frivolity's Labours

**Published:** 1890-08-16

**Authors:** 


### Passing Topics.

### FRIVOLITY'S LABOURS.

We must not begrudge the hard-working journalist his annual holiday. The leading article writer, notwithstanding the portentous impersonality which he assumes in the English "dailies"—the blending of power with incorporeality—is but mortal, and, perchance, is frail. In any case he needs his rest like other busy persons in this workaday age. It has been said that the writers upon any given newspaper attain, after the probationary period, to a more or less uniform style of thought and diction. It is certainly true that the man who reads one morning paper regularly, in time adopts its method of thinking, and is nervously sensible of any departure from its usual courses. This being so, editors ought to be careful not to inflict upon their constant and confiding readers literary pabulum which, swallowed in trust, cannot be assimilated without suffering and a grave dis-arrangement of the digestive processes.

We are led into these observations by the perusal of an article in the *Standard*, which as completely as any we have lately met with illustrates our complaint. Inspired by the approach of Goodwood races, and evidently desirous of ex-hibiting the orthodoxy of August by a seasonable worship of idleness, the writer consumes nearly a column of space in depicting the labours of frivolous people with a view to introduce the knotty question—"but, after all, what is frivolity?" As if conscious this is a poser, he proceeds to enlighten us by predicating that "probably everything is more or less frivolous that is not downright hard work, ill-paid for"—a curious proposition which puts us in mind of nothing more readily than the aspiration of the lazy man who devoutly wished that lying on the grass in the summer was reckoned as hard work and well remunerated.

But what it most behoves us to protest against in these columns is a previous hazard of this imaginative writer, intro-duced, moreover, by the pious ejaculation—"let us be just." "There is many a London lady who is supposed to do nothing from morning to night but amuse herself, but who is in reality as hardworked as any public man or any one of these shop-girls and linendrapers' young men, concerning whose ex-cessively long hours of labour there periodically arise loud and prolonged wailings."

We ask seriously whether the reasoning is worthy of the reputation of a great journal, even in the dog days? If ever obvious fallacy existed, it exists here, and we only wonder that in his search for examples our writer did not bethink himself to include hospital nurses.

What, we wonder, have the early closing associations to say to the prodigious proposition that a lady of fashion who at her own sweet will and amid luxurious surroundings has enjoyed Ascot and Henley, Lord's and the Opera, with numerous dances, dinners, and garden parties, and when this precious article appeared was about to spread her wings for Goodwood and then to the sea or the mountains for the glorious autumn-tide, is as hard worked as the weary, footsore shopgirl and drapers' assistant with their one week's holiday in the year?

Of course, what the *Standard* means is that pleasure is pursued and enjoyed until it ceases to be all it once was. But though pleasure and idleness be taken to satiety, they remain pleasure and idleness still. To argue to the contrary is as sensible as to say that beef-steak ceases to be beef-steak when you have eaten enough of it.

It is bad enough, though inevitable, that such inequalities exist. They are among the concomitants of the artificiality we call civilization, and which confers among many benefits much that is cruel. The summer sun is a benevolent factor, but, nevertheless, it helps to vitalize, if it do not breed, many noxious organisms. Civilization is sunshine, but from an ideal world we should banish its paramours—fashion and society, together with all parasitic noodles who claim for their votaries the dignity of labour.

Agricola.

